# Editorial: Language embodiment, volume II: interdisciplinary methodological innovations

**DOI:** 10.3389/fpsyg.2023.1251549

**Published:** 2023-08-11

**Authors:** Laura M. Morett

**Affiliations:** ^1^Department of Educational Studies in Psychology, Research Methodology, and Counseling, University of Alabama, Tuscaloosa, AL, United States; ^2^Department of Speech, Language and Hearing Sciences, University of Missouri, Columbia, MO, United States

**Keywords:** language embodiment, research methods, event-related potential (ERP), eye-tracking, self-paced reading, transcranial magnetic stimulation, neuropsychology, emotion

This Research Topic expands upon the Research Topic *Language Embodiment: Principles, Processes, and Theories for Learning and Teaching Practices in Typical and Atypical Readers*. Like its predecessor, this Research Topic maintains that the body plays a central role in influencing language processing (Glenberg and Kaschak, 2003). In particular, this Research Topic showcases methodologically innovative work on diverse topics providing support for embodiment in language processing.

Real-time measures of reading provide insight into ease and depth of processing for text conveying content consistent with embodied experience in comparison to text conveying content inconsistent with embodied experience. Using self-paced reading, Liao et al. examined processing of the innovative *Bei* construction in Mandarin, which intrinsically activates negative constructional meaning via embodied social grounding. Phrases conveying the negative constructional meaning of the innovative *Bei* construction were read more quickly than phrases conveying the partial literal meaning of the innovative *Bei* construction and unrelated phrases, indicating that its comprehension is facilitated via construction-based processing. Using eye-tracking, Wang et al. investigated English cataphora resolution by Chinese English as a foreign language (EFL) learners by varying the gender of the first potential antecedent. Reading times for the first potential antecedent were longer when it was congruent than incongruent in gender with the cataphoric pronoun. These results provide evidence that Chinese EFL learners prioritize lexical over syntactic cues in English sentence comprehension, indicating increased reliance on embodied experience. Jia et al. used eye-tracking and key logging to examine the influence of anxiety on translation to and from English in Chinese EFL learners. Both the eye-tracking measures of fixation count and duration and the key logging measures of pause count and duration were higher for forward (Chinese-English) than for backward (English-Chinese) translation, and fixation duration and pause count were positively correlated with Beck Anxiety Inventory scores for both directions of translation. Finally, Zheng and Wang used eye-tracking to probe the influence of positive affect from pun comprehension on ongoing cognitive processing. Although reading times for salient homophones were read faster in congruent control sentences than less salient homophones in sentences with homophone puns, regressions from the homophone and sentence final regions were more likely in congruous control sentences than sentences with homophone puns, and sentence reading time was negatively correlated with humor ratings. Taken together, the results of Jia et al. and Zheng and Wang provide evidence that embodied affect exerts a powerful influence on language processing, consistent with the tenets of embodied theories of language processing (Foolen, 2012).

Valence categorization provides insight into the impact of explicit embodied affective processing on language comprehension. Zhang and Fan used a priming paradigm to illuminate the effects of hot (i.e., emotion-related) and cold (i.e., non-emotion-related) executive function on language switch costs during Chinese-English emotional word categorization. Larger language switch costs were observed when primes and targets were congruent rather than incongruent in emotion and for negative than positive emotional words when primes and targets were congruent in emotion, as well as for participants with high than low inhibitory control for negative target words congruent with primes. Tang et al. compared categorization of emotion-label (i.e., words labeling emotional states) and emotion-laden (i.e., words evoking emotions via connotation) words in Chinese and English by Chinese-English bilinguals. Facilitated processing of emotion-label over emotion-laden words in both Chinese and English was observed, and only negative English emotion-laden words were not processed more quickly than neutral words. Together, these findings of these two studies provide further evidence of the strength of the influence of embodied affect on language processing, providing additional support for embodied theories of language processing.

Event-related potentials (ERPs) provide evidence of implicit sensitivity to content eliciting embodied experience conveyed via language that may not be evident in behavioral measures such as responses and reading times. Gu et al. investigated how new words learned with connotations of disgust and sadness expressed via faces are integrated into sentences conveying disgust and sadness. A larger negative waveform was observed for sad than disgusting words in the 146–228 ms time window, whereas a larger positive waveform and greater current densities in emotion- and language-related brain structures were observed for emotionally congruent than incongruent trials in the 304–462 ms time window. Shen et al. compared processing of Chinese and English scientific metaphors by Chinese-English bilinguals. Relative to Chinese scientific metaphors, English scientific metaphors elicited a larger N400, a smaller late positive component (LPC), and a larger late negativity. Cao et al. examined processing of pictorial metaphors in conjunction with their verbalizations. A larger posterior P300 was observed for pictorial juxtapositions in conjunction with verbal metaphors of the structure A is like B and for literal images in conjunction with verbal metaphors of the structure A is B than for other combinations. Considered as a whole, these findings provide new insight into the neural bases of emotion and metaphor processing, contributing to the substantial and growing literature on this topic (Buccino et al., 2016).

Neurostimulation and neurological disorders provide causal evidence for the role of neural structures in embodied language processing. Vitale and de Vega used low-frequency repetitive transcranial magnetic stimulation to demonstrate that primary motor cortex, but not primary visual cortex, subserves memory for action language processing. Phillips et al. examined comprehension and production of tactile metaphors by an individual born without somatosensation and found that it was comparable to that of individuals with typical somatosensation. These findings challenge strong views of embodied cognition positing that direct sensory experience enables somatosensory simulation, which is necessary to comprehend and produce tactile metaphors. Together, these studies demonstrate how causal data permits conclusions about how the brain enables simulation of embodied experience during language processing.

In summary, the articles in this Research Topic leverage a variety of innovative methods to provide evidence of the body's influence on language processing, complementing other recent work that does so (Tian et al., 2020; Morett et al., 2021; Herbert, 2022). In doing so, they contribute to the advancement of the understanding of embodied cognition, particularly with respect to language processing, as well as the methodological repertoire used to advance it. Thus, they provide methodological inspiration for future research on embodied language processing, broadening the methodological scope used to reveal how sensorimotor experience influences written and spoken language processing.

## Author contributions

The author confirms being the sole contributor of this work and has approved it for publication.
